# Automated computer-assisted quantitative analysis of intact murine lungs at the alveolar scale

**DOI:** 10.1371/journal.pone.0183979

**Published:** 2017-09-21

**Authors:** Goran Lovric, Ioannis Vogiatzis Oikonomidis, Rajmund Mokso, Marco Stampanoni, Matthias Roth-Kleiner, Johannes C. Schittny

**Affiliations:** 1 Centre d’Imagerie BioMédicale, École Polytechnique Fédérale de Lausanne, 1015 Lausanne, Switzerland; 2 Swiss Light Source, Paul Scherrer Institute, 5234 Villigen, Switzerland; 3 Institute for Biomedical Engineering, ETH Zurich, 8092 Zurich, Switzerland; 4 Institute of Anatomy, University of Bern, 3012 Bern, Switzerland; 5 Max IV Laboratory, Lund University, SE-221 00 Lund, Sweden; 6 Clinic of Neonatology, University Hospital of Lausanne (CHUV), 1011 Lausanne, Switzerland; University Children’s Hospital Bern, SWITZERLAND

## Abstract

Using state-of-the-art X-ray tomographic microscopy we can image lung tissue in three dimensions in intact animals down to a micrometer precision. The structural complexity and hierarchical branching scheme of the lung at this level of details, however, renders the extraction of biologically relevant quantities particularly challenging. We have developed a methodology for a detailed description of lung inflation patterns by measuring the size and the local curvature of the parenchymal airspaces. These quantitative tools for morphological and topological analyses were applied to high-resolution murine 3D lung image data, inflated at different pressure levels under immediate *post mortem* conditions. We show for the first time direct indications of heterogeneous intra-lobar and inter-lobar distension patterns at the alveolar level. Furthermore, we did not find any indication that a cyclic opening-and-collapse (recruitment) of a large number of alveoli takes place.

## Introduction

Synchrotron-based lung imaging techniques with small animals (rats, mice, rabbits) have been established in various studies and provided insights into some of the most interesting questions in lung physiology and development [1–3]. Broadly speaking, three complementary approaches have been pursued with success: (i) static 3D imaging of fixed lung samples with spatial resolutions down to one micrometer have been routinely used for characterizing in high detail various aspects of individual rat lung acini and their developmental stages [4–7]; (ii) dynamic *in vivo* radiographic (2D) studies at pixel sizes down to 10 micrometers for investigating lung liquid clearance phenomena, effects of positive end-expiratory pressures (PEEP) and improved ventilation strategies [8–10], with recent advances towards 3D [11] and with Fourier space signature analysis [12]; (iii) *in vivo* low spatial resolutions (with pixel sizes of 30 *μ*m and above) tomography [13, 14] has been applied to study various alterations to regional gas distributions in the lung, such as the effect of PEEP [15], tidal volume [16] or the heterogeneity introduced by particular disease models [17, 18]. Recent advances in fast X-ray tomographic microscopy [19] allow to anticipate that tomographic *in vivo* imaging of the lung at the micrometer scale is within reach [20, 21]. In contrast to these experimental developments, little has been improved in the quantitative analysis of high-resolution 3D lung images. The standard structural analysis of the lung is still mostly based on stereology, which may be unsuitable for handling high-throughput data and for establishing an automated (computer-guided) process [22, 23].

From a pure post-processing point of view, lung tissue comprises a binary structure of air and tissue, analogous for instance to the one of solidified alloy materials [24, 25]. During lung development, the continuous optimization process of increasing gas-exchange surface in accordance with lung volume growth results in a very complex hierarchical structure with a huge air-to-tissue surface area [26–28]. Hence, two intrinsic quantities are affected for the most part, the air-to-tissue surface area with its shape and the air volume within the lung. To investigate small structural changes in lung tissue at the micrometer scale, it is thus necessary to assess these quantities with high fidelity in 3D, posing requirements for both experimental realization as well as post-processing from segmentation to quantification. While the detailed air recruitment mechanism is still under debate [29], a full quantitative characterization of lung tissue could bring new facts to light, supporting either of the two currently discussed hypotheses: (i) a heterogeneous distention pattern of different lung areas [30] or (ii) a homogeneous cyclic opening-and-collapse (recruitment) of all alveoli [31].

In this work we present a full route to quantitative analysis of high-resolution 3D lung image data, starting from the image acquisition scheme for intact animals, how it particularly affects the segmentation and by making the link to quantitative 3D characterization of lung tissue. We employ local structural thickness analyses for assessing volumetric changes of air volumes at various structural scales. For the topological analysis of the air-to-tissue surface in the lung, we apply the theory from differential geometry to calculate localized surface curvatures. We show for the first time the results of air volume thickness map and curvature analyses performed on dose-efficient fast tomographic images of intact lungs. Great attention is paid to keeping the methods as descriptive as possible as they are released as part of the manuscript under General Public License and can easily be expanded and applied to a wide range of examples coming from different disciplines.

## Materials and methods

### Image acquisition

**Fig 1:**
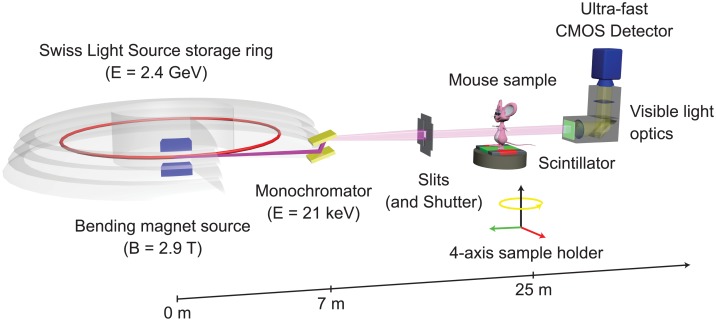
Experimental setup. Experimental setup at the X02DA TOMCAT beamline, with permission from [20].

The experiment was carried out at the X02DA TOMCAT beamline of the Swiss Light Source (SLS) at the Paul Scherrer Institute (Villigen, Switzerland). The experimental setup is adapted from one of our previous works [20] and depicted in Fig 1: the X-ray beam, produced by a 2.9T bending magnet on a 2.4GeV storage ring (with ring current *I* = 400mA, top-up mode), is monochromatized with a double-multilayer monochromator and tuned to 21keV. A sample-to-source distance of 25m is used for producing an X-ray beam with appropriate spatial coherence properties. We used a high-speed CMOS detector (pco.Dimax) coupled to visible-light optics with a 150 *μ*m and 20 *μ*m thick scintillator for medium and high spatial resolutions, respectively. The samples were probed with two different optics, yielding effective pixel sizes of 2.9 × 2.9 *μ*m^2^ and 1.1 × 1.1 *μ*m^2^, respectively. For these two optics the field of view was adjusted with horizontal and vertical slits, located just before the sample, and producing beam sizes of 5.8 × 2.7 mm^2^ and 2.2 × 2.2 mm^2^, respectively. The sample-to-detector distance *z* was set to 100 mm, yielding an optimal trade-off between contrast-to-noise ratio and resolution at the respective experimental settings [20]. All scans were performed with 5 ms single-projection exposure times and 901 tomographic projections, giving total scan times in the range of 5 s. The photon flux on the sample position was measured using a high-precision passivated implanted planar silicon (PIPS, Canberra Industries Inc.) diode coupled to a multi-gain low-current amplifier that was previously calibrated by cryogenic radiometry, achieving precisions with less than a few percent uncertainty [21]. From the flux measurement, the entrance dose *D* is calculated according to the following formula [32]:

$$\begin{array}{c} D\;\text{[Gy]}=1.602\times 10^{-4}\cdot \frac{I_{0}\;[\text{photons}/\mu {\text{m}}^{2}]\cdot h\nu \;\left[\text{eV}\right]}{\mu^{-1}\;[\mu \text{m}]\cdot \rho \;[\text{g}/{\text{cm}}^{3}]}, \end{array}$$ (1)

For all scans the entrance dose per projection (modeled for water) was 0.56 Gy for the respective field of view.

### Animal preparation

The measurements were performed *post mortem* on adult mice (*n* = 2 / Balb-C, central animal facility of the University of Bern) that were sacrificed before the experiment. The mice were anesthetized with an injection of a mixture of Fentanyl, Midazolam and Medetomidine. Subsequently, a tracheotomy was performed and the animal was placed, together with the endotracheal cannula, in an upright position into a custom-made sample holder. Immediately prior to imaging, an overdose of pentobarbital was administered to the animal in order to prevent motion artifacts caused by the heart beat. The lung was then inflated to different pressures (10 cmH_2_O, 20 cmH_2_O and 30 cmH_2_O) using a small-animal ventilator (FlexiVent, SCIREQ Inc.), and for each pressure level a separate tomographic scan was performed. Images were taken no longer than 30 min after the lethal injection to preserve quasi-*in vivo* conditions. All parts of the animal experiments were approved and supervised by the Swiss Agency for the Environment, Forest and Landscape and the Veterinary Service of the Canton of Bern.

### Post-processing

The aforementioned setup facilitates propagation-based phase-contrast images, which were input to the single-image phase and intensity extraction algorithm by Paganin *et al*. [33] and subsequently CT-reconstructed with the *gridrec* algorithm [34]. However, this acquisition scheme—coupled with the low-exposure and low number-of-projections setting—produces CT reconstructions with various artifacts that make an automatic segmentation impossible. The reason is that the single-image phase retrieval enhances edge-blurring, leading to an overall decrease in resolution as well as to a reduction of visibility of very thin structures [20]. Although this effect can be overcome by using a simple fusion algorithm that combines the phase-retrieved images with the absorption ones to yield enhanced edge contrast, it reduces the signal-to-noise ratio in the reconstructed images and thus makes an automatic segmentation in low-exposure images more challenging [35]. Therefore, in the present work we have focused on an improved alternative post-processing approach. We first discuss the aforementioned image artifacts before describing our segmentation method in detail.

**Fig 2:**
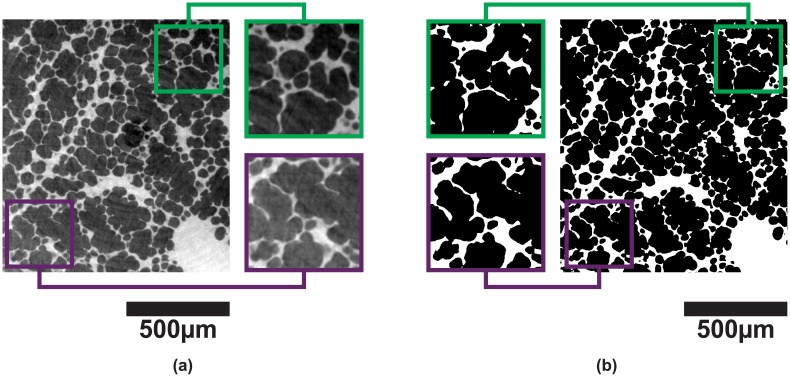
Threshold segmentation. Result of automatic threshold segmentation: (**a**) Original tomographic slice (1.1 × 1.1 *μ*m^2^ pixel size); (**b**) binary image after automatic Otsu segmentation. The background illumination gradient produces different segmentation results for the two regions in the tomographic slice.

As shown in Fig 2, most dominantly, different regions in the lung produce varying segmentation results, while other effects are not immediately obvious. For instance, in Fig 2a the thin septal walls that separate single alveoli produce only a slightly stronger signal than the surrounding background. Additionally, the region-of-interest (“local” or “truncated”) tomography introduces a superimposed gradient in the grayscale images resulting in different foreground (lung tissue) and background (air) gray values for different regions in the tomographic slices [36], leading to the fact that in Fig 2b the interalveolar septa are not correctly recognized for the two regions. Finally, the differentiation of foreground and background is strongly dependent on the inspected lung region itself (not shown here), leading to an additional per-slice variation of background illumination gradients and tomographic slice histograms. Thus, in the best case several steps are necessary for obtaining the binary segmented images of lung tissue and air which in return serve as a basis for further quantitative analyses. For addressing all the above issues we have developed a semi-automatic technique that is applicable to any comparable problem in image segmentation. The method is automatized to the full extent, except in one single step, where it requires user interaction in order to decide which segmentation result is sufficient for further data processing.

**Fig 3:**
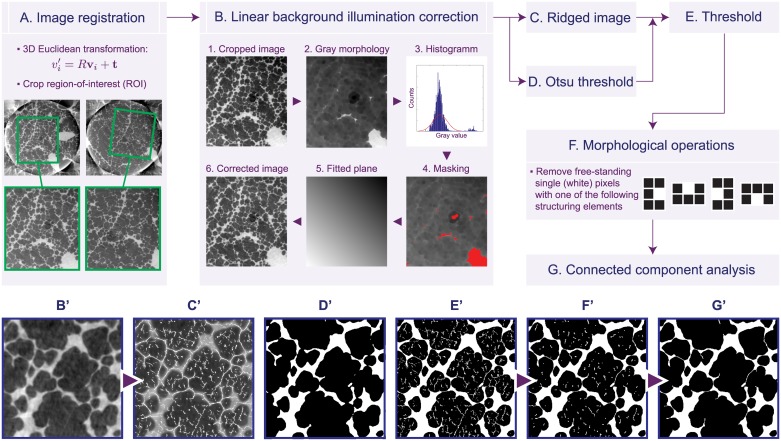
Segmentation flowchart for 3D lung data. Complete segmentation flowchart from image registration to final binary segmentation of the high-resolution lung data. Image registration (**A**) is conducted by calculating a rotation matrix *R* and a translation vector *t* and by transforming all image voxels; in (**B**) from each cropped tomographic slice an estimated linear gradient image is subtracted (**B.5**); the ridged image (**C’**) is obtained by means of line-shaped profiles; the images are thresholded (**E’**) with the Otsu-value (**D**); finally the morphological operations (**F**) and the connected component analysis (**G**) remove noise artifacts. The result is best visible by comparing image **D’** to image **G’**.

The detailed post-processing flowchart is depicted in Fig 3 and the processing steps can be summarized as follows:

1. First [Fig 3(A)], the datasets at different peak-inspiratory pressures are registered manually with each other to compensate for shifts in the sample position and the irregular up-scaling of the air volumes upon inflation. As a result, we obtain cropped gray-level images of regions of interest of the lung tissue, which are related to each other under a Euclidean transformation. This means, for all volumes the 3D fields of view have the same sizes.
2. In the next step [Fig 3(B)], background illumination correction is applied to correct the gradient arising from the region-of-interest (local) tomography artifact, as discussed above. This represents a common problem in image processing, and various morphological operations have been suggested, each adapted to particular problems [37]. In our case we assume that the background is a plane (i. e. decribed by a linear gradient) and we perform a so-called erosion (with a disk-shaped structuring element of 20 px). Erosion represents one of the two fundamental operations in morphological image processing and can be thought as follows: an image is probed, pixel by pixel, with a structuring element which defines a per-pixel neighborhood; in the case of a disk-shaped structuring element, each pixel’s neighborhood has a circular shape with a given radius; erosion of a given pixel then means that its respective gray-scale value is exchanged with the smallest gray-scale value within the whole neighborhood. In theory, when applied to all pixels in the image, the background gradient is then clearly visible, but also strongly structure-dependent. For this reason, all extreme values are removed from the histogram creating a so-called “masked” image. Subsequently a plane is fitted by means of least-squares onto the masked image and in the last step subtracted from the original one. For the choice of the (size of the) structuring element, it is important to be large enough in order to bring the background gradient into prominence, however, a very large structuring element also increases computing speed. In our case, a structuring element of 20 px was sufficient for “filtering out” thin structures (such as alveolar septa) and at the same time for maintaining an adequate computation speed.
3. Automatic Otsu segmentation [38] is applied [Fig 3(D)] and the optimal gray value is stored for latter usage. This step is only used for determining the threshold (gray value).
4. In parallel [Fig 3(C)], a so-called “ridged image” is created based on the idea of so-called line-shaped profiles, originally introduced by Babin *et al*. [39]. The idea is motivated by the following fact: In the original image thin septa are visible by eye as they slightly differ from the background, but their signal is too weak to be recovered during an automatic (global) segmentation step. If we now take a line profile through the image and extract all local maxima from this line profile, in theory we should be able to recover each septa. By introducing further mathematical constraints, this step could be refined in order automatically “accept” or “decline” a certain structure to be recognized as a septum. In practice, this step is the only one that requires user interaction in order to let the user decide the margin for septa discrimination from the background. In our algorithm we enable the discrimination of septa by defining a minimal/maximal width, and minimal and maximal gray-value margin in respect to the background. The line profiles are conducted in four directions in the image: 0°, 45°, 90° and 135°. Whenever a septum is detected, the respective pixel value in the original image is set to the brightest value, making sure that from now the (alleged) septum will be always detected as tissue. However, as seen in Fig 3(C’), due to noise in the background, new features (i. e. artifacts) are added as well.
5. In the next step [Fig 3(E)], binary threshold is conducted on the ridged image [Fig 3(C’)] by using the Otsu parameter from (3).
6. Morphological operations [Fig 3(F)] are applied to remove free-standing pixels, since (4) has produced (dependent on the explicit conditions) a significant number of artifacts. Here we use four manually-defined simple structuring elements that are based on the following assumption: a single free-standing pixel *always* represents an artifact because septa (being very thin and sometimes occupying only single pixels) are connected with surrounding lung tissue.
7. Finally [Fig 3(G)], a connected component analysis is conducted in 3D to remove artifacts that are thicker than single pixels and that were not removed during the previous steps. After this step the final binary segmented image is used for further processing.

### Local air volume thickness analysis

**Fig 4:**
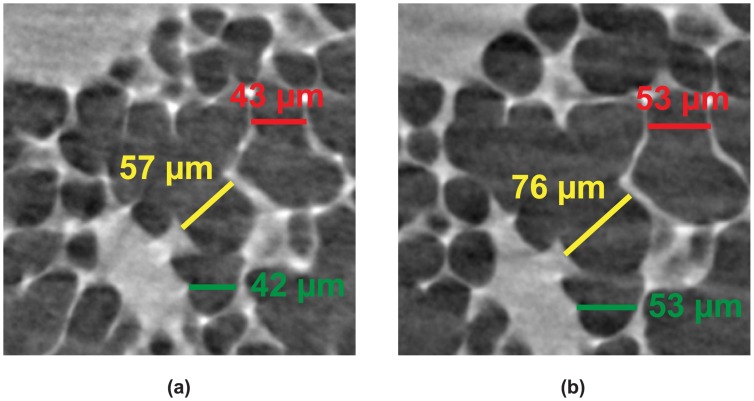
Air volume thickness. Manual measure of air volume “thickness” on a 2D tomographic slice at two different peak inspiratory pressures: (**a**) for 5 cmH_2_O; (**b**) for 10 cmH_2_O.

The volumetric change of alveolar structures upon inflating the lung with increasing peak-inspiratory pressures in intact animals has been measured by different means so far, either by manual counting [40] or by detecting changes in the power spectral density of lung speckle images [41]. These measures, however, provide only limited insight. Either they are acquired at the lung periphery areas or they only give global volume changes without insight into the detailed processes (i. e. with no direct observations). In Fig 4, only a small part of the lung is illustrated to show how lung tissue stretches at two different pressures. Our aim in the following is to automatically quantify these volumetric changes in 3D. Similar tasks are usually performed in various other studies, such as bone [42] or materials science [43], for which a so-called thickness map analysis has been developed to determine the thickness of trabecular bone [44]. The local thickness *τ* is defined as follows:

$$\begin{array}{c} \tau \left(p\right)=2\times \text{max}\;(\{r|p\in \text{sph}(x,r)\subseteq \Omega ,x\in \Omega \}), \end{array}$$ (2)

where **p** is an arbitrary point of a set Ω, Ω ⊂ ℝ^3^ defines the 3D structure under study, sph(**x**, *r*) is a sphere with center **x** and radius *r*. As can be seen from that formula, *τ* simply gives the maximum diameter of the spheres that fit inside an arbitrarily chosen object. In our case, in order to get the diameter of the airspaces we define the air volume as the “object”, which must not be confused with tissue thickness of the lung parenchyma. The detailed calculation steps are then as follows: at first, the Euclidean distance map is calculated, from which a so-called ridge (or skeleton) of the distance map is extracted; afterwards, the ridge is scanned in order to find the largest possible sphere that the ridge belongs to; as a final step, a so-called cleanup is applied on the surface voxels. A detailed description of all steps can be found in the original source code [45]. As a result, one obtains a 3D dataset where the respective local air volume thickness values *τ* are mapped onto the gray values of every voxel. A simple histogram then yields the *relative amount* of structures with a certain size that exist in the 3D image data, i. e. the distribution of structural size diameters [44].

Comparing thickness map histograms (distributions of structural size diameters) under different conditions with each other, in our case thickness maps of lung volumes that are inflated at different pressures, can be problematic due to the fact that histogram bins can be chosen arbitrarily and may be influenced by outliers and noise. In order to overcome this problem, kernel density estimation comes in handy as an often used technique in non-parametric data smoothing and quantitative density comparisons. In the following work we adapt the kernel density estimator by Duong [46], implemented in the *R* statistics package [47].

### Curvature analysis

The second part of the quantitative analysis was conducted on the air-to-tissue interfaces in the lung. While the local air volume thickness gives a pure local volumetric representation, it does not provide information on the topological properties of the material (such as surface normals, surface facet areas etc.). Topology in the form of varying surface curvatures is commonly studied in the field of material science, in particular with alloy structures, where it represents an important factor providing indication of local variations in surface energy [24] or gives the direction of diffusion-driven flux [25]. In biological materials such as plants structure curvature has been found to be genetically controlled [48], along with other biophysical perspectives of this quantity. Recently, the potential in medical image analysis has been recognized, but the full application remains still in its beginning phase [49, 50].

The mathematical background originates from the theory of differential geometry, for which we mainly follow the descriptions in [51]. In brief, to study the properties of regular surfaces in 3D it is convenient to define the so-called *shape operator* at a point p∈M, where M represents a regular surface in ℝ^3^. It is defined as the linear transformation *S* of the tangent space $M_{p}$ that measures how M bends in different directions. In practice, this is achieved by further defining a nonzero vector $v_{p}\in M_{p}$ to determine the direction of the surface bending while the real-valued function for doing so is called the *normal curvature*. The minimum and maximum values of the normal curvature, *κ*_1_ and *κ*_2_, are then called the *principal curvatures* and, by further mathematical treatment, it can be shown that these correspond to the eigenvalues of the aforementioned shape operator. Additionally, the principle curvatures are directly linked to the mean curvature *H* and the Gaussian curvature *K* by

$$\begin{array}{c} \kappa_{1}=H-\sqrt{H^{2}-K}\;\text{and}\;\kappa_{2}=H+\sqrt{H^{2}-K}, \end{array}$$ (3)

following the intrinsic algebraic properties of the shape operator.

From a computational point of view, surfaces are represented by (polygonal) meshes, which are described by a collection of vertices, edges and faces that define the surface shape in 3D. While there exist many different representations of polygon meshes, usually the simplest one is the so-called *face-vertex* mesh representation which consists of a list of vertices (3D position vectors) and a set of polygons (commonly: triangles) that point to the vertices they encompass. Since any polygonal mesh represents a discretization (and thus approximation) of a smooth surface, a number of methods have been proposed to yield curvature estimations on such objects [52]. In the present work, we employ a curvature estimator based upon the theory of normal cycles [53] to associate a curvature tensor with each region on the polyhedral surface. This approach is particularly useful when dealing with sampled smooth surfaces, because it is possible to define a small neighborhood for a given vertex which provides the curvature tensor in the smooth case at the same vertex [53]. We explain this aspect below in more detail and note at this point that in practice the method yields fast and very precise curvature estimations of large datasets.

**Fig 5:**
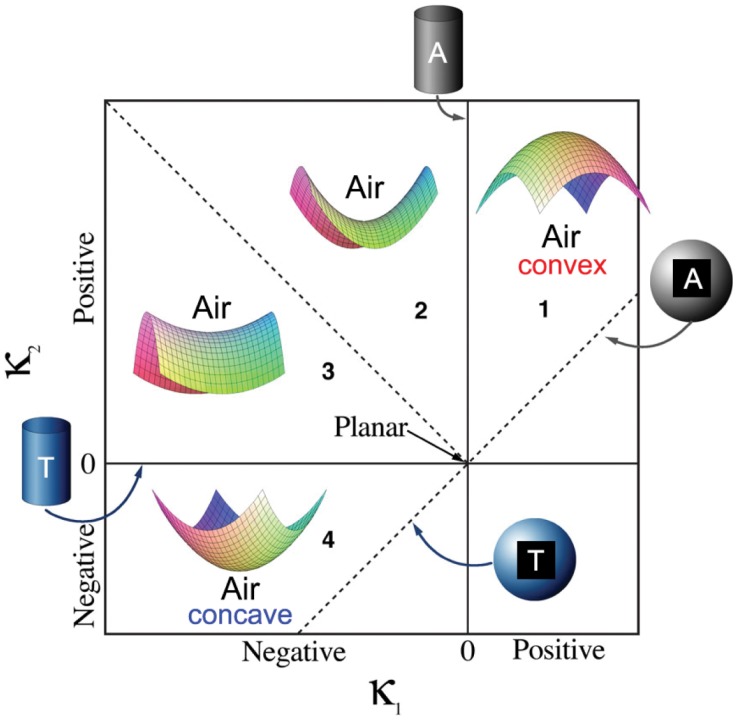
Interface shape distributions map. Interface shape distributions (ISD-plot) schematic, adapted with permission from J.L. Fife [25]. It represents a 2D probability density plot in dependence on the the minimum curvature *κ*_1_ and the maximum one *κ*_2_ for each vertex on the mesh. It gives a graphical representation of how interfaces are shaped within the investigated 3D volume. The four regions (**1–4**) are explained as follows: *κ*_1_, *κ*_2_ > 0 (**region 1**) represent ellipsoidal surfaces (being convex toward the lung tissue); *κ*_1_, *κ*_2_ < 0 (**region 4**) indicate concave ones; and **regions 2** and **3** represent so-called hyperbolic (or saddle) surfaces. The color code (blue = concave, red = convex) of **regions 1** and **4** is the same as in Fig 13, while “A” and “T” denote cylindrical/spherical surface shapes of “air” and (lung) “tissue”, respectively.

Based on the per-vertex curvature calculations of the whole 3D dataset, we introduce the so-called ISD (interface shape distribution) plots, which are two-dimensional probability density functions in dependence on the two principal curvatures *κ*_1_ and *κ*_2_. We adopt here the *κ*_1_–*κ*_2_ representation from [54], for which an example is shown in Fig 5. As can be seen, the ISD plot gives a graphical representation of the overall curvature information within the investigated 3D dataset. In terms of mesh orientation, it means that we demand the surface normals on the polygon mesh to point from the air volume toward the lungs tissue. Thus, following regions can be identified: *κ*_1_ = *κ*_2_ > 0 represent spherical shapes of the air volume while *κ*_1_ = *κ*_2_ < 0 indicate spherical shapes of the lung tissue; cylindrical shapes are characterized by *κ*_1_ = 0 or *κ*_2_ = 0; *κ*_1_, *κ*_2_ > 0 (region 1) represent ellipsoidal surfaces (being convex toward the lung tissue) while *κ*_1_, *κ*_2_ < 0 (region 4) indicate concave ones; finally, regions 2 and 3 represent so-called hyperbolic (or saddle) surfaces.

**Fig 6:**
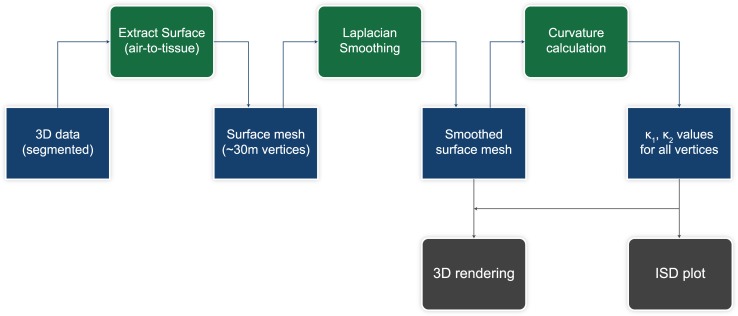
Curvature analysis flowchart. Processing flowchart for the curvature analysis. From the segmented volume the surface is first extracted [55], creating a surface mesh of about 30 million vertices. Subsequently Laplacian smoothing [56] is applied to reduce high-frequency noise, and curvature calculation is performed [53] to extract the two principal curvatures for all vertices. These are then used to create the ISD-plots and the 3D renderings.

We end the section by describing the detailed processing pipeline, as displayed schematically Fig 6:

1. Starting from the 3D segmented data, the air-to-tissue surface mesh is first created with the so-called marching-cubes algorithm, implemented in VTK [55]. The produced triangulated mesh, depending on the original data size, can have up to 30 millions of vertices.
2. High-frequency noise originating from discrete data is then removed by Laplacian smoothing [56], implemented in the open-source MEPP platform [57]. The noise occurs due to sharp borders of single pixels.
3. The principle curvatures are then calculated on the smoothed surface mesh from a curvature estimator algorithm based on normal cycles [53], also implemented in the MEPP platform [57]. The aforementioned vertex neighborhood is set by means of a geodesic radius.
4. Finally the data is further processed to create either 3D renderings according to the principal curvatures or calculate 2D probability densities, the so-called ISD plots. For the latter ones we apply the aforementioned kernel density estimator [46].

## Results

### The quality of binarized images

**Fig 7:**
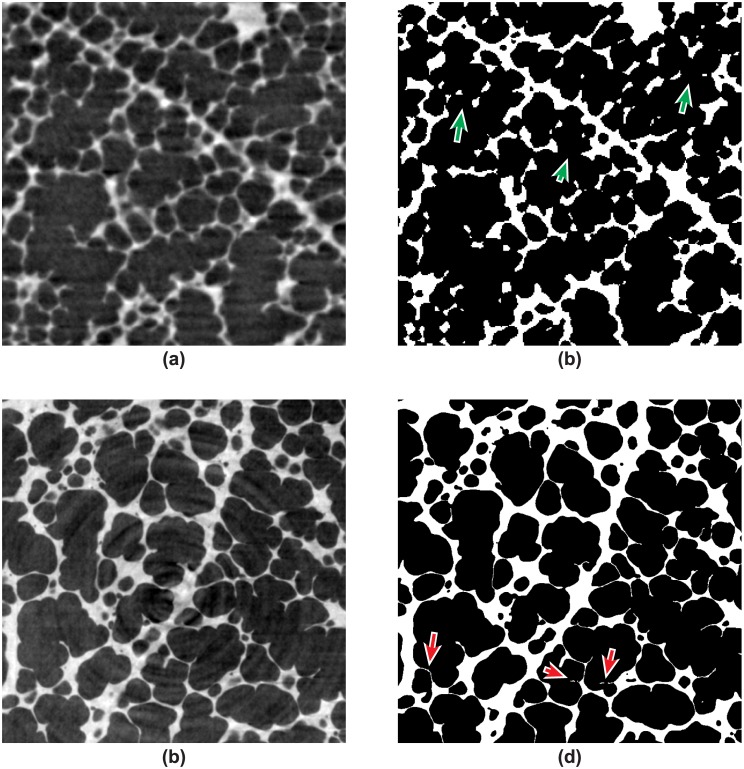
Low-resolution vs. high-resolution optics. Comparison between the low-resolution and high-resolution optics for a 0.8 × 0.8 mm^2^ field of view on two randomly chosen regions: (**a**) shows the tomographic slice of the 2.9 *μ*m-pixel-size optics; (**b**) the corresponding binary segmented image; (**c**) shows the tomographic slice of the 1.1 *μ*m-pixel-size optics; and (**d**) shows its corresponding segmented image. The arrows display artifacts introduced by the segmentation.

Tomographic slices of the lung tissue of two *post mortem* (intact) animals imaged with both the 2.9 *μ*m-pixel-size and the 1.1 *μ*m-pixel-size optics are plotted in Fig 7. The slices are both cropped to cover an area of 0.8 × 0.8 mm^2^. In the lower-resolution raw images in Fig 7a the thin walls between the alveoli (septa) are visible, but are lost in the segmentation step [as indicated by the green arrows in Fig 7b]. With the high-resolution optics, the septa can be recovered in the binary segmented image [as shown in Fig 7d] by applying our segmentation technique. However, small artifacts are introduced when conducting the “ridged-image” step in the segmentation method (step C in Fig 3), which are indicated with the red arrows in the segmented image [see Fig 7d]. Since both the high- and low-resolution optics under low-exposure conditions produce image segmentation artifacts, we need to shortly discuss their implications in view of the subsequent quantitative analysis.

For the lower-resolution images [Fig 7a and 7b], the almost complete vanishing of septal surfaces during the segmentation step leads to the fact that for a topological analysis of the gas-exchange surface area (the alveoli) crucial data is missing. Thus, the low-resolution data appear unsuitable for a quantitative curvature analysis. For the air volume thickness map analysis, however, we hypothesize that the artifacts will only play a marginal role since they are expected to produce only single-pixel alterations in the localized airspace volumes. Hence, the only remaining part is the one of finding a “sufficiently” well-segmented volume before inputting the data to further analysis. Distinguishing between a “good” and “bad” segmentation can sometimes be ambiguous, as the distinction can be made by different criteria (biological features, SNR, etc.). To overcome this problem, we first produced an automatized binarized 3D dataset [38] and independently applied a morphological “opening” and “closing” operation ending up with a total of three datasets per peak-inspiratory pressure. By this means, the following quantitative results become independent on the segmentation step because we only need to define a range of different segmented volumes that we consider valuable in terms of preserving the main biological features. As we describe in the next section, the results of the quantitative analysis then possess quantifiable uncertainties originating from possible segmentation errors.

The higher-resolution images, as seen upon visual inspection from Fig 7c and 7d, appear suitable for both the air volume thickness map as well as the curvature analysis. Again, the visible artifacts are expected to play only a marginal role on the air volume thickness map analysis while topologically they represent “sharp” surfaces (with small radii) which can be easily filtered upon topological (curvature) evaluation. To investigate the influence of different binary image segmentations, the parameters that require user interaction in our segmentation method (i. e. the ones that specify the detection of local maxima in the line-shaped profile algorithm—step “C” in Fig 3) were manually varied to produce 9 datasets for each peak-inspiratory pressure, resulting in datasets which have varying degrees of visible artifacts. In particular, these were the minimum/maximum width for discriminating alveolar septa in the respective line profile as well as the minimum gray-value threshold that defines alveolar septa in respect to the background. Subsequently, all datasets were input to the quantitative analysis algorithms.

### Air volume thickness map results

**Fig 8:**
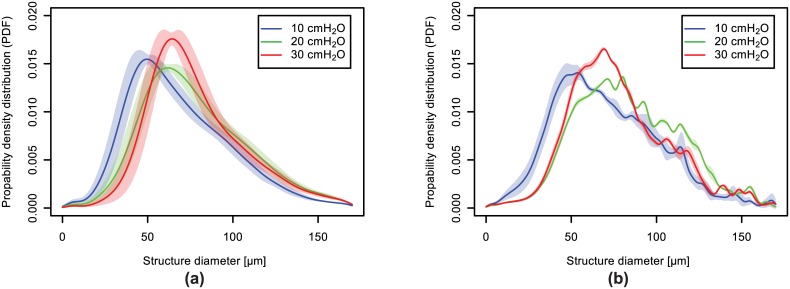
Local air volume thickness PDF. Probability density functions (PDF) of the local air volume thicknesses for the two different optics: (**a**) shows the air volume thickness-map PDF for the 2.9 *μ*m-pixel-size optics; (**b**) shows the one for the 1.1 *μ*m-pixel-size optics. The uncertainty intervals arise from the different sets of segmentations.

**Fig 9:**
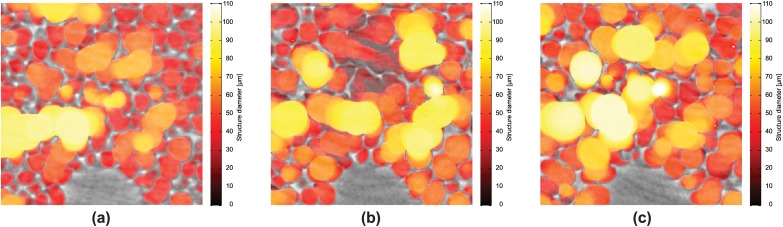
Structure diameters. Structure diameters obtained by air volume thickness map calculation with the 1.1 *μ*m-pixel-size optics for three different peak inspiritory pressures: (**a**) 5 cmH_2_O; (**b**) 10 cmH_2_O; (**c**) 25 cmH_2_O.

As mentioned before, multiple binarized 3D datasets per peak-inspiratory pressure (3 different segmentations for the low-resolution optics and 9 different segmentations for the high-resolution one) were created for both optics in order to investigate the influence of the segmentation step on the quantitative results. This is reflected in Fig 8 and Table 1, while the air volume thickness map visualizations (both 2D and 3D) were conducted only on one selected segmentation per inflation pressure. One such example is shown in Fig 9 for small regions of interest, where the air volume thickness maps for every single peak-inspiratory pressure are superimposed on the original data. The colors are mapped in respect to the structural diameters. As it can be seen, with increasing pressure, there is an increase of orange-to-yellow structures corresponding to structural diameters of approximately 70 *μ*m and a decrease of red structures, corresponding to structural diameters of about 40 *μ*m.

**Table 1:** Volumetric distributions of the thicknesses at different ranges and magnifications. The results are obtained by integrating the PDF-s from Fig 8 according to the corresponding ranges.

Pressure	Range 1 (20 − 50) *μ*m	Range 2 (50 − 80) *μ*m	Range 3 (80 − 110) *μ*m	Range 4 ≥ 110 *μ*m
**10 cmH_2_O** (2.9 *μ*m)	(28.7 ± 6.4)%	(37.2 ± 3.0)%	(21.0 ± 2.3)%	(10.2 ± 1.8)%
**20 cmH_2_O** (2.9 *μ*m)	(16.4 ± 5.5)%	(40.2 ± 3.1)%	(25.5 ± 2.8)%	(15.8 ± 2.6)%
**30 cmH_2_O** (2.9 *μ*m)	(12.3 ± 5.8)%	(46.3 ± 5.9)%	(25.3 ± 4.0)%	(14.4 ± 2.3)%
**10 cmH_2_O** (1.1 *μ*m)	(25.4 ± 4.0)%	(35.8 ± 1.5)%	(23.6 ± 2.4)%	(11.7 ± 3.7)%
**20 cmH_2_O** (1.1 *μ*m)	(11.8 ± 0.6)%	(36.0 ± 0.7)%	(29.6 ± 0.7)%	(20.4 ± 1.2)%
**30 cmH_2_O** (1.1 *μ*m)	(13.5 ± 1.3)%	(43.9 ± 1.1)%	(25.6 ± 1.0)%	(14.7 ± 2.0)%

In Fig 8 the probability density functions (PDF) of the air volume thickness maps are plotted in dependence on the structure diameters for the two different optics. The plots have been limited to structural air volume thicknesses of up to 170 *μ*m due to the simple fact that big volumes can alter the results when the lungs inflate as they move in/out of the region of interest. The colored areas behind each function display the standard deviations of the quantitative results in respect to the different segmentation parameters used for the calculations. From the curves, we observe a shift from small diameters (around 40 *μ*m) at a peak-inspiratory pressure of 10cmH_2_O towards bigger diameters (around 70 *μ*m) with increasing pressure. This result is observable with both optics, and there appears to be no significant change in the calculated local air volume thickness distributions between the two optics. However, the data that was acquired with the higher magnifying optics [see Fig 8b] yields significantly more precise (with smaller standard deviations) results than the lower magnifying one [as seen in Fig 8a]. Furthermore, the increase of the interpulmonary pressure from 10 cmH_2_O to 20 cmH_2_O results in a distinct enlargement of the parenchymal airspace, but the further increase from 20 cmH_2_O to 30 cmH_2_O shows only a minimal enlargement. The latter might indicate that the total lung capacity is reached at an interpulmonary pressure of roughly 20 cmH_2_O.

In Table 1, the volumetric distributions at four different ranges (20 − 50 *μ*m, 50 − 80 *μ*m, 80 − 110 *μ*m and 110–rest *μ*m) are summarized. They are obtained by integrating the respective distributions over the given intervals and show the same trends in a quantitative manner: with increasing peak-inspiratory pressure the volumetric increase happens at *Range 2* with a simultaneous decrease at *Range 1*.

As can be seen both from Fig 8 and Table 1, the results from both optics exhibit matching trends for the two smaller ranges *Range 1* and *Range 2*. The structural diameters above 80 *μ*m, on the other hand, produce probability densities that do not follow an unambiguous trend with increasing peak-inspiratory pressures. We explain this effect later in more detail, but note at this point that this is due to the small volumetric regions of interest that introduce additional biases. Structural diameters of less than 20 *μ*m are not regarded in the evaluation process as they are much smaller than the smallest expected diameters of the alveoli.

**Fig 10:**
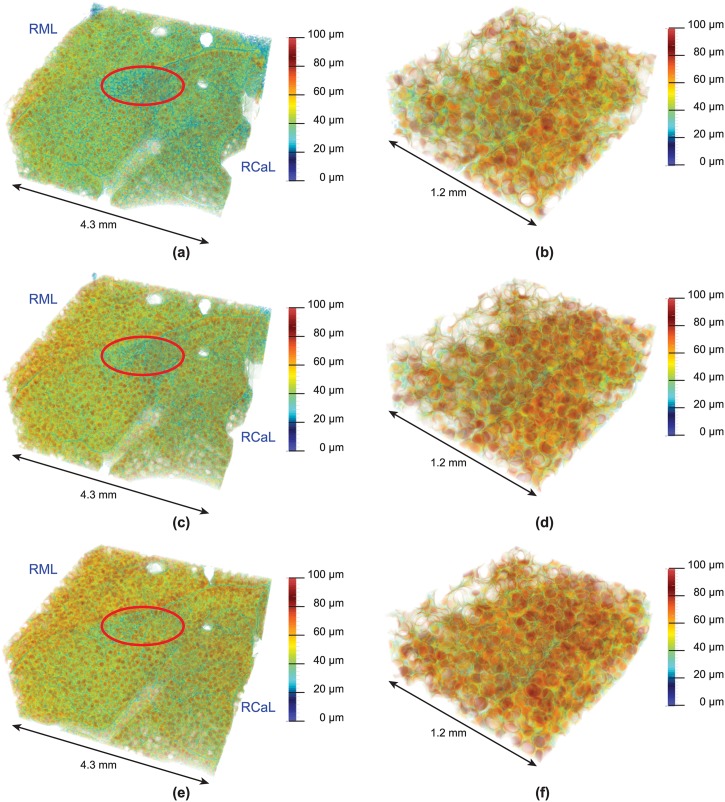
3D air volume thickness maps. Visualization of the air volume thickness maps in 3D: (**a**),(**c**) and (**e**) show the 10, 20 and 30 cmH_2_O pressures for the 2.9 *μ*m-pixel-size optics; (**b**),(**d**) and (**f**) show the ones for the 1.1 *μ*m-pixel-size optics. RML: right middle lobe; RCaL: right caudal lobe; the red circles indicate areas which at the same air pressures exhibit a smaller expansion of the individual airspaces. With higher pressures more orange-to-red colored volumes are visible, i. e. air volumes with structural thicknesses of 50 − 80 *μ*m.

Finally, in Fig 10 we show 3D representations of the air volume thickness maps for the three different pressures and the two optics. In the low-resolution optics (bigger field-of view) the big airways have been excluded by being transparent (visible by the holes). The line running roughly from the lower left to the upper right corner of the block represents the border between the right middle and right caudal lobe, and it appears that the middle lobe increases more in volume than the caudal one, following the color representation. This is noticeable through the fact that the middle lobe adopts a more “reddish” color with bigger inflation. Therefore, a heterogeneous inflation was observed both inter- and intra-lobular.

### Curvature results

**Fig 11:**
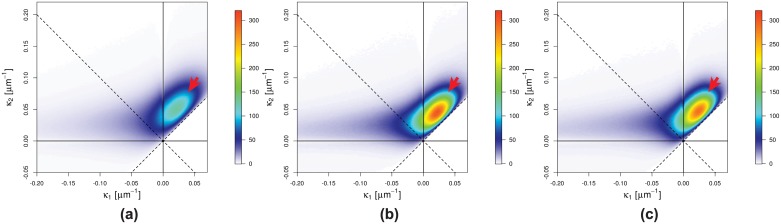
ISD plots. Interface shape distributions (ISD) for the free different pressures: (**a**) 10 cmH_2_O; (**b**) 20 cmH_2_O; (**c**) 30 cmH_2_O. The arrows indicate the shift towards smaller principle curvatures (larger radii).

The interface shape distributions (ISD) for the three different pressures are shown in Fig 11. Since for each peak-inspiratory pressure multiple ISD-s are calculated in respect to the different binarized datasets obtained using nine different sets of parameters for the segmentation, here the mean ISD-s are plotted. As expected, the highest density lies in “region 1”, compared to the ISD-definition plot in Fig 6 and indicates that lungs are largely ellipsoidaly shaped (i. e. convex toward the lung tissue), similar to the ideal shape of alveoli [58]. It is further visible that with increasing pressures a transformation from a diverse to a more uniform distribution of curvatures on the air-to-tissue surface takes place. This is visible with the emergence of the bright (red) peak after the increase of the interpulmonary pressure from 10 to 20 cmH_2_O. The last step (from 20cmH_2_O to 30cmH_2_O) becomes even more localized in “region 1”. However, the increase from 10cmH_2_O to 20cmH_2_O produces a much larger difference than the increase from 20cmH_2_O to 30cmH_2_O. Again this is due to the fact that the total lung capacity is reached at roughly 20cmH_2_O. At the same time, the blue tail (density ∼ 90) from “region 2” and “region 3” (Fig 11) slightly shifts toward the central peak (right direction). In total, however, the highest-density peak moves towards smaller principal curvatures (as indicated by the arrow in Fig 11) or larger radii, respectively. This is slightly visible by the move of the upper “blue-violet tail” (density ∼ 70) toward the center.

**Fig 12:**
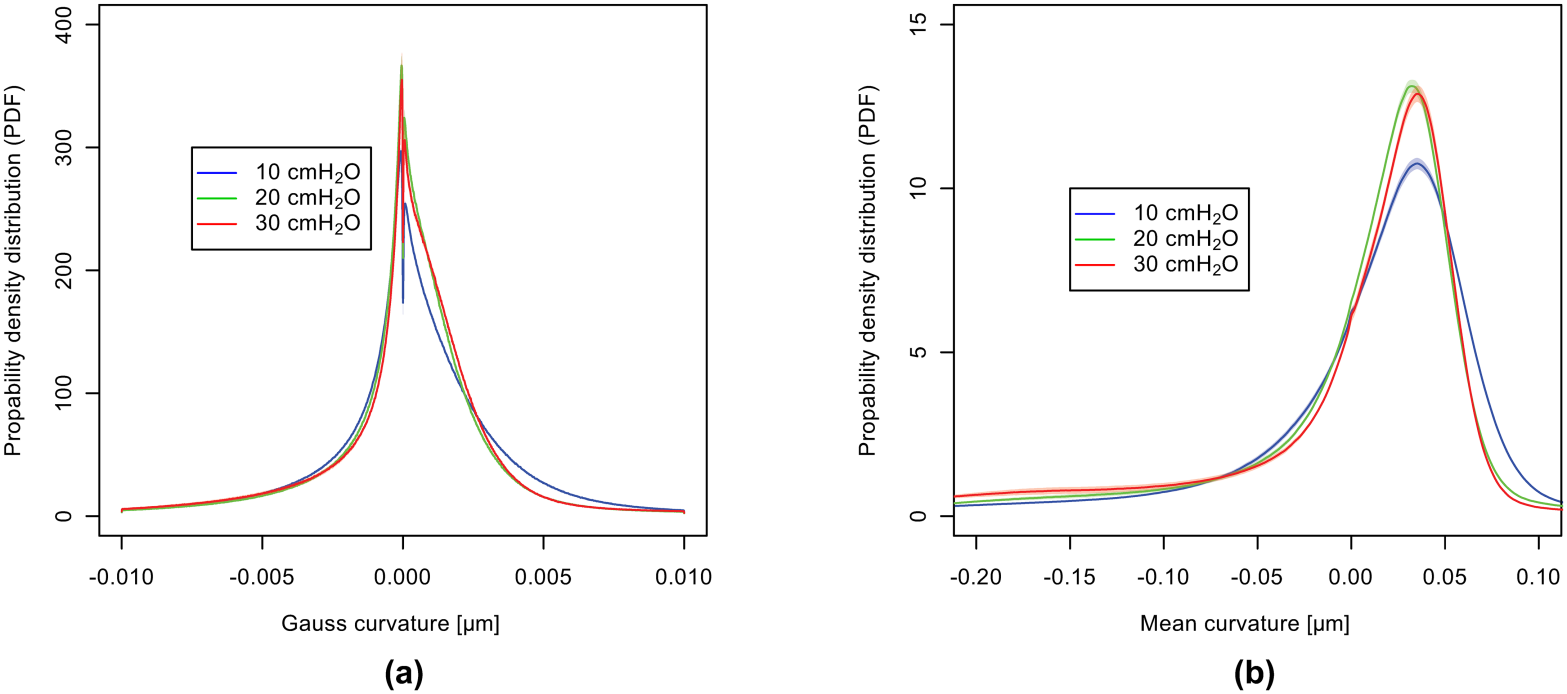
Gaussian and mean curvatures PDF. Probability density functions (PDF) of Gaussian (**a**) and mean (**b**) curvatures. Like before, the uncertainty intervals arise from the different segmentation parameters.

To better clarify these results, in addition both the Gaussian and the mean curvatures for the different pressures are plotted in Fig 12. As before, the colored areas behind each function display the standard deviations of the quantitative results in respect to the different segmentation parameters. As can be seen from Fig 12, the error margins are very small indicating that the different segmentation parameters have very little influence on the overall curvature results.

The Gaussian curvature is best illustrated by a flat surface such as an expanding disk that grows isotropically [48]: if the expansion is uniform (i. e. the overall shape remains the same) it will have zero Gaussian curvature; if the marginal regions grow slower than the central ones, the disk will exhibit a parabolic shape and the Gaussian curvature will be positive; and if the central region grows slower than the marginal ones, the disk will buckle and form a shape with a wavy edge (e. g. a saddle surface), rendering a negative Gaussian curvature. In our case, we observe an increase around zero, indicating that the existing surfaces within the lung *only* become more flat, as can be seen in Fig 12a. On the other hand, a slightly higher density is visible for positive Gaussian curvatures, indicating again the presence of ellipsoidal (convex toward the lung tissue) surfaces.

The mean curvature as shown in Fig 12b indicates the aforementioned trend (with increasing peak-inspiratory pressures) towards smaller principle curvatures in “region 1”, meaning that positive mean curvatures in the outer right regions become flatter. The fact that the peak in Fig 12b shows an inverted trend from 20cmH_2_O to 30cmH_2_O can be attributed to the same effect that was observed already in the air volume thickness map analysis. Namely, since we are considering only small partial volumes of intact lungs, big airways can move out of the volumetric field-of-view upon higher peak-inspiratory pressures, which is why the results for low mean curvatures (i. e. flat surfaces, big airways) have to be considered with care.

## Discussion

The presented analyses were performed on tomographic volumes acquired in rapid scanning mode optimized for dose versus image contrast. Our purpose was to keep the total exposure time and radiation dose at minimum in order to render the analyses relevant also for potential future *in vivo* measurements. Under these conditions, the produced volumes require more effort for being transformed into binarized datasets, which on the other hand represents the starting point for any further quantitative analysis. Therefore, under these conditions, it can hardly be determined whether a segmentation is unambiguously “correct”, especially in the presence of obvious image artifacts. This fact was addressed by performing the quantitative analyses on multiple binarized images per each original dataset (three datasets for each lower-resolution image set and 9 datasets for each higher-resolution image set). The margin for producing these different binary segmentations was defined by visual inspection so that apparent “under”- and “over”-segmented volumes where created. These were then all input to the quantitative analysis. By doing so, single-pixel errors originating from the binary segmentation were directly translated to the uncertainty of the quantitative results.

From the results of the air volume thickness maps we identify structural diameters of 50–80 *μ*m to increase in density when increasing the peak-inspiratory pressure in the lung. The results are comparable at both scales, meaning for both the low-resolution and the high-resolution optics and indicate that the existing structures (20 − 50 *μ*m) increase their volume, which is why they vanish with higher peak-inspiratory pressures (visible in Figs 8 and 9 and Table 1). The observed sizes are similar to the ones reported in other studies [59, 60], even if a comparison to the alveolar size is not directly possible, because the thickness map measures airspaces which contain both alveolar airspaces and parts of the alveolar ducts. In the 3D visualizations of the air volume thickness maps (Fig 10), two observations were made. First, the airspaces do not increase in their size evenly in all areas of the lung parenchyma. For instance, the encircled regions in Fig 10 at the same pressures depict smaller enlargements of the individual airspace volumes when compared to the neighboring ones. Secondly, comparing two lobes (right middle and caudal lobe) it appears that their inflation requires different pressures, too. Therefore, we conclude that the pulmonary inflation follows an intra- and interlobar heterogeneous distension pattern. The probability density function of the air volume thicknesses obeys the same distribution and mostly describes an isometric up-scaling of alveolar structures (visible by the shift to the right direction upon increasing inflation pressures). A homogeneous cyclic opening-and-collapse of all alveoli was postulated as part of the normal ventilation of the lungs [31]. If, however, a large number of alveoli would open during inhalation, a non-isometric up-scaling of the alveolar structure should be visible in the probability density function of the air volume thickness maps. This would mean an increase of alveolar structures of low volumes and hence smaller structural size diameters (as they just popped-up), in difference to a homogeneous right deviation of the density curve towards higher volumes. Because these morphological observations have only been conducted on partial volumes of the whole lung, we cannot exclude a smaller amount of alveoli (1–20%) showing “opening-and-collapse” behavior during the breathing cycle. Therefore, we propose that a cyclic opening-and-collapse of the alveoli does not take place to a larger extend.

As mentioned earlier, for structural diameters above 80 *μ*m the results increasingly differ between the two optics. This is mainly attributed to the fact that with higher magnifications a smaller fraction of the lung volume is analyzed. Hence, airways outreaching the borders of the field of view seemingly produce densities with smaller structural diameters in the air volume thickness map analysis. This bias is even more pronounced for smaller fields of view and when bigger airways are being analyzed. For a truly valid analysis it would thus be necessary to conduct the analysis on individually anatomical features encompassing complete acini and/or lung lobes.

The findings from the curvature analysis are complementary, namely that lungs are mostly described by ellipsoidal (convex toward the lung tissue) shapes (*κ*_1_, *κ*_2_ > 0) that grow in diameter with increasing pressure. This fact is best seen by looking at the interface shape distribution (ISD) plots in Fig 11, where the highest-density peak is located in “region 1” (*κ*_1_, *κ*_2_ > 0), as well as by looking at the positive mean curvature values in Fig 12b. Interestingly, from Fig 12b it can also be seen that the density of high negative mean curvature values (−0.20 ⩽ *H* ⩽ −0.07 *μ*m^−1^) increases with increasing pressure while the one of low negative mean curvature values (−0.07 ⩽ *H* ⩽ 0 *μ*m^−1^) decreases, which is attributed to the behavior of saddle surfaces within the lungs. The Gaussian curvature from Fig 12a clearly suggests that all surfaces are becoming more flat towards higher pressures, which would not have been the case if a larger number of new small alveoli would open upon inflation. In terms of lung anatomy, Gaussian curvature can also be interpreted as follows: in the case of a broad distribution we have saddle-surfaces and sharp edges (free septal edges or alveolar mouths, respectively), ellipsoidal (alveoli) and spherical shapes etc. If the distribution becomes sharper around zero it means that there is a trend in all these surfaces towards a more flat shape, which obviously can only be the case for alveoli.

The curvature *per se* also plays a further role. The alveolar surface tension is not only regulated by pulmonary surfactant but also dependent on the local curvature, where a higher curvature results in a higher surface tension. For instance, surface tension is expected to be greater in smaller alveoli and directly related to dissipative energy [61]. Therefore, a measurement of the local curvatures may contribute to a better understanding of differences in local surface tensions during any alteration of the size of the alveoli, be it during breathing or during lung development [62].

**Fig 13:**
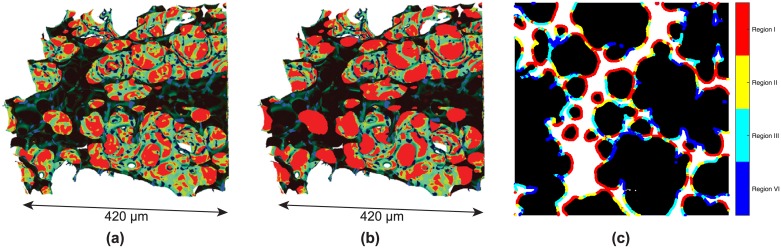
Different geodesic radii. 3D visualization of the four regions from the ISD-plot with different geodesic radii: (**a**) depicts the calculation with radius *R* = 3.5 and (**b**) with *R* = 15. In (**c**) a 2D slice is shown from (**b**) where the mesh vertices are mapped back to the gray-level image.

**Fig 14:**
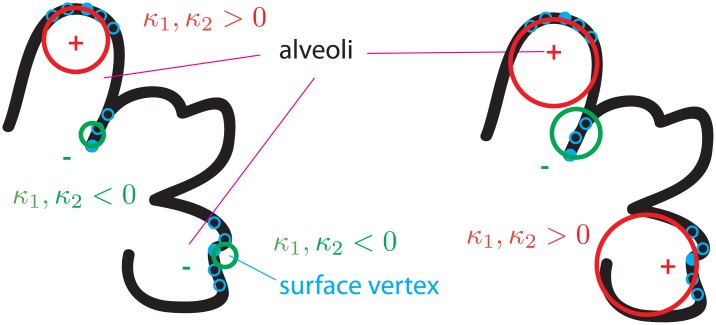
Sketch of alveolar areas. Sketch for depicting how small areas within the alveoli can be “forced” to be recognized as alveolar area, despite having a different shape. On the left side, the red circle (corresponding to the geodesic radius) identifies an ellipsoidal shape, while the green ones identify negative ellipsoidal shapes. If the geodesic radius is increased, the vertex area in the lower right alveolus will be recognized as belonging to the alveolar surface.

Since we found that the predominant part of the lung air-to-tissue surface is described by ellipsoidal shapes (convex toward the lung tissue), we shortly address the question whether a curvature analysis is enough to uniquely identify alveolar surfaces within the lung. This question is relevant because it could help defining a unique anatomical and topological model for alveoli, which usually exhibit very diverse geometric shapes [63]. For this purpose, the four regions of the ISD-plot (Fig 5) are visualized in 3D in Fig 13a. As it can be seen, the red surfaces (corresponding to “region 1”) are indeed shown to lie on alveolar surfaces, however there are small surface areas that have different shapes (non-ellipsoidal) in between, which again corresponds to previous findings in lung anatomy indicating that the overall shape of the alveoli is very irregular [61, 64]. The apparent question is now, how could non-ellipsoidal surfaces that are part of alveolar surfaces be distinguished from others that are not (septal edges or alveolar mouths). This can be achieved by making use of a simple assumption when applying the normal cycle algorithm for calculating curvatures. Namely if we set a big geodesic radius at each surface vertex small areas lying in the middle of the alveoli which do not have a strict ellipsoidal surface will be interpreted as ellipsoidal due to their surroundings. This fact is illustrated in Fig 14: first (on the left side), the curvatures are mimicked to be calculated with small geodesic radii; then (once the geodesic radius increases) small deviations in the alveolar surface area are still recognized as being part of an alveolus. The result is plotted in Fig 13b and its corresponding 2D tomographic slice is shown in Fig 13c. The detailed treatment of whether this approach alone suffices the unique identification of alveolar surfaces, however, goes beyond the scope of the present work which is why we note it here for discussion.

## Conclusion

We have presented a technique for the fast acquisition of 3D lung image data from intact *post mortem* animals, followed by a detailed post-processing scheme ranging from segmentation to quantitative analysis. In doing so, we applied two established quantitative measures to characterize fresh lung tissue at high resolutions and in 3D, in particular the air volume thickness map [44] and curvature analysis [25], which to the best of our knowledge represents the first evaluation of high-resolution lung data of this kind. All presented tools are published as a complete package under the GNU General Public License and available for download at the TOMCAT homepage [65]. Hence, our post-processing and evaluation technique can serve as a complete toolbox for characterizing and analyzing lung data at the alveolar and acinar scale. Applied to the lung samples, we found first indications for a heterogeneous intra-lobar and inter-lobar distension pattern, although still only observed *post mortem* in intact animals. The results further indicate that a cyclic opening-and-collapse of alveoli (a recruitment of alveoli during inhalation) does not take place at a large extend during breathing. First indicative results are presented showing that the curvature analysis might serve as a tool for automatically identifying alveolar surfaces in high-resolution 3D lung image data. This could play a role in finding a unique geometrical description of an alveolus, which in return could be applied for an automatic counting of alveoli. Finally, since our tools are completely based on open-source tools/algorithms, they can easily be expanded and applied to a wide range of other disciplines, materials and studies.
